# Effects of diet-induced obesity and voluntary wheel running on the microstructure of the murine distal femur

**DOI:** 10.1186/1743-7075-8-1

**Published:** 2011-01-17

**Authors:** Hongqiang Ma, Tuomas Turpeinen, Mika Silvennoinen, Sira Torvinen, Rita Rinnankoski-Tuikka, Heikki Kainulainen, Jussi Timonen, Urho M Kujala, Paavo Rahkila, Harri Suominen

**Affiliations:** 1 Department of Health Sciences, University of Jyväskylä, Finland; 2 Department of Physics, University of Jyväskylä, Finland; 3 Department of Biology of Physical Activity, University of Jyväskylä, Finland

## Abstract

**Background:**

Obesity and osteoporosis, two possibly related conditions, are rapidly expanding health concerns in modern society. Both of them are associated with sedentary life style and nutrition. To investigate the effects of diet-induced obesity and voluntary physical activity we used high resolution micro-computed tomography (μCT) together with peripheral quantitative computed tomography (pQCT) to examine the microstructure of the distal femoral metaphysis in mice.

**Methods:**

Forty 7-week-old male C57BL/6J mice were assigned to 4 groups: control (C), control + running (CR), high-fat diet (HF), and high-fat diet + running (HFR). After a 21-week intervention, all the mice were sacrificed and the left femur dissected for pQCT and μCT measurements.

**Results:**

The mice fed the high-fat diet showed a significant weight gain (over 70% for HF and 60% for HFR), with increased epididymal fat pad mass and impaired insulin sensitivity. These obese mice had significantly higher trabecular connectivity density, volume, number, thickness, area and mass, and smaller trabecular separation. At the whole bone level, they had larger bone circumference and cross-sectional area and higher density-weighted maximal, minimal, and polar moments of inertia. Voluntary wheel running decreased all the cortical bone parameters, but increased the trabecular mineral density, and decreased the pattern factor and structure model index towards a more plate-like structure.

**Conclusions:**

The results suggest that in mice the femur adapts to obesity by improving bone strength both at the whole bone and micro-structural level. Adaptation to running exercise manifests itself in increased trabecular density and improved 3D structure, but in a limited overall bone growth

## Background

Bone strength/quality is not only determined by bone mineral density/mass alone but also by its geometrical structure and distribution in space. Both peripheral quantified computed tomography (pQCT) and micro-computed tomography (μCT) have thus been used in recent years for rodent skeleton measurements. Since the first application of pQCT to the mouse skeleton density measurement was reported in 1996 [1], it has been frequently used to measure the volumetric bone mineral density and geometrical parameters of trabecular and cortical bone in vivo or ex vivo. However, due to its limited resolution, pQCT has failed to obtain information on the trabecular microstructure. The introduction of μCT with at best a submicrometer resolution in biomedical research has made accurate assessment of the material microstructure possible. Based on different algorithms, the 3D structure and related parameters of bone can be obtained. They describe the properties of bone better than the "golden standard" histomorphometry based on stereological assumptions.

Osteoporosis and obesity are two common complex diseases with serious health-related consequences. These two disorders of the body composition have usually been considered separately, but more recently both clinical and experimental data have established a close link between them [2]. Epidemiological data and animal studies indicate that these two diseases share several features including a genetic predisposition and a common progenitor cell [3], and both are influenced by nutrition and a sedentary life style [4]. Obesity is a condition of excessive body fat that causes or exacerbates the risk for developing non-insulin dependent diabetes, cardiovascular diseases, cancer, and other diseases [5], and is associated with chronic inflammatory status [6]. Osteoporosis is a disease characterized by low bone mass and structural deterioration of bone tissue, leading to bone fragility and increased susceptibility to fractures.

Nutrition, including dietary fat [7] and restricted caloric intake [8], are related to molecular markers of bone remodelling and may contribute to the risk for bone-related diseases [9]. Diets high in saturated fat can adversely affect bone mineralization [10]. Diet components are also closely associated with obesity in both humans [11] and animals [12]. A recent genetic linkage analysis between obesity and osteoporosis has found evidence of genetic influences behind these two disorders being related and likely mapped to many of the same quantitative trait loci [13]. These linkages have also been documented in previous epidemiological investigations, where obesity has been associated with bone mass, strength and density [14,15]. Furthermore, there is abundant evidence that adipose tissue, as an endocrinal organ, affects bone metabolism through secreted adipokines, especially leptin [16,17]. Together with secreted inflammatory factors, these adipokines alter the bone microenvironment and regulate bone modelling and remodelling. However, whether these relations are positive or negative remains controversial.

In obese subjects, physical activity has been shown to be an efficient tool to treat obesity-related diseases and improve the quality of life, and also to improve bone quality in both humans [18] and animals [19]. Consistently, our previous study with twin pairs showed that long-term leisure time physical activity has positive effects on both trabecular and cortical bone [20]. In mice, voluntary wheel running was associated with increased trabecular bone mineral density [21].

Despite the complex relationships between bone, obesity and physical activity, it is clear that both obesity and physical activity affect bone metabolism. The purpose of this study was to determine how diet-induced obesity combined with voluntary exercise affects cortical and trabecular bone properties. We measured these bone properties by high resolution μCT together with pQCT measurements on the distal femoral metaphysis.

## Methods

### Animals and Diets

This study was approved by the National Animal Experiment Board, Finland. Forty 6-week-old male C57BL/6J mice were obtained from Taconic (Ejby, Denmark). The mice were housed, one per cage, in a humidity- and temperature-controlled room with a 12:12 light cycle (08.00:20.00), and allowed to adapt to their new environment for 1 week before being allocated to one of the four intervention groups: control diet (C), control diet + voluntary running (CR), high-fat diet (HF), and high-fat diet + voluntary running (HFR).

To study the effect of voluntary wheel running exercise, animals in the CR and HFR groups were housed in custom-made cages with a running wheel (diameter 24 cm, width 8 cm) to which they had free access 24 h/day for 21 weeks. Total wheel revolutions were recorded daily by a magnetic switch, with the total exercise performed per day determined by multiplying the number of wheel rotations by the circumference of the wheel. C and HF animals were housed in similar cages without the running wheel. The mice had continuous access to the control or high-fat diet, respectively, and to regular tap water. Body mass and food consumption were measured at two-week intervals. The control diet was a standard rodent diet, R36 (4% fat, 55.7% carbohydrate, 18.5% protein, 3 kcal/g, Labfor, Stockholm Sweden). The high-fat diet was a lard-based purified diet, D12492 (60% fat, 20% carbohydrate, 20% protein, 5.24 kcal/g, Research Diets, Inc., USA).

### Glucose Tolerance and Insulin Resistance Tests

For all the mice glucose and insulin tolerance tests (GTT and ITT, respectively) were performed at one-week interval after 10 and 18 weeks of intervention. Briefly, mice were fasted for 5 h before GTT and 2 h before ITT. Glucose (2 g/kg body mass) or insulin (0.75 U/kg BM; Humuline^® ^Regular, Eli Lilly, Indianapolis, IN) solution was injected into the intraperitoneal cavity, followed by blood sampling from the shaved hind limb vein at time points 0, 15 (ITT only), 30, 60, 90, and 120 min. Glucose level was determined by a B-Glucose photometer (HemoCue AB, Angelholm, Sweden). Total area under the GTT (AUC-GTT) and above ITT (AUC-ITT) curves was calculated using the trapezoidal rule.

### Serum Biomarkers

After 21 weeks of intervention, the mice were sacrificed by cervical dislocation. Blood samples were collected and sera separated after clotting for 1 hour and stored at -70°C for further analysis. Concentrations of insulin, leptin, osteoprotegerin (OPG), osteocalcin, resistin, and plasminogen activator inhibitor-1 (PAI-1) in serum were measured using the Milliplex mouse bone metabolism panel (Millipore, Bedford, MA) according to the manufacturer's instructions.

### Specimen Collection

After the mice were sacrificed, the left femur was separated and trimmed of attached soft and connective tissues, wrapped within PBS-soaked gauze, and stored frozen at -20°C. Liver and epididymal fat pads were surgically removed.

### pQCT Densitometry

**Figure 1:**
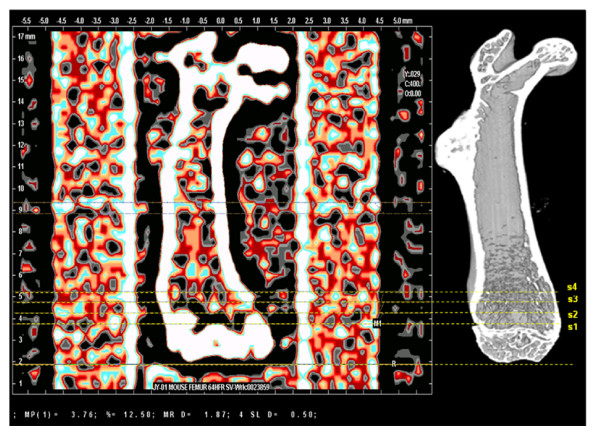
**pQCT (left) and μCT (right) overviews of a mouse femur**. The position of slices (s1 - s4) scanned by the pQCT are shown. The same volume of interest was analyzed also by μCT.

The femur was thawed overnight at 4°C and inserted into a specially constructed plastic syringe with the shaft in the axial direction. Scanning was done with a pQCT apparatus (Stratec XCT Research SA, Stratec Medizintechnik GmbH, Pforzheim, Germany) calibrated using a hydroxyapatite standard cone phantom. A voxel size of 0.07 × 0.07 × 0.5 mm was used in all the measurements. The scout view was obtained from the entire bone for landmark detection (see Figure 1). Four slices (s1-s4) were scanned at 0.5-mm intervals starting from 12.5% landmark. All the scanned slices were analyzed by bone analysis software (Geanie 2.1, Commit, Espoo, Finland). A threshold value of 500 mg/cm^3 ^was used for the separation of trabecular and cortical bone. From s4, total bone circumference (CfB), total bone cross-sectional area (CSA), total bone mineral density (BMD) and content (BMC), density-weighted maximum (Imax), minimum (Imin), and polar moment of inertia (Ipolar) were determined. Cortical cross-sectional area (cCSA), cortical bone mineral density (cBMD) and content (cBMC), cortical thickness (ThC: using a ring model), trabecular cross-sectional area (traCSA), trabecular bone mineral density (traBMD) and content (traBMC), and marrow cross-sectional area (mCSA) were also assessed. Compressive strength index (CSI = BMD^2 ^× CSA) was determined. After the pQCT measurements, the distal half of the femur was stored at -4°C and fixed with 70% ethanol for further analysis. All pQCT measurements and data analyses were performed by the same individual. The coefficients of variation (CV) for repeated measurements were 2.0%, 0.5%, 2.8%, and 1.2% for cTh, cBMD, traBMD, and CSA, respectively.

### μCT Measurements

μCT is an imaging method that produces (in the absorption mode) a 3D density map of the sample at very high spatial resolution. The left distal femur was scanned with a Skyscan 1172 desktop μCT scanner (Skyscan, N.V., Aartselaar, Belgium). The X-ray source was set to 90 kV and 112 μA, with a voxel size of 2.8 μm. The projection images were acquired over an angular range of 360° with an angular step of 0.4° and reconstructed using a cone-beam reconstruction software based on the Feldkamp algorithm yielding 1600 cross-sectional images. Four regions (s1, s2, s3, and s4) (Figure 1) were selected. Each region contained 174 cross-sectional images corresponding to the thickness measured by pQCT. For the cortical shell and trabecular structure analysis, we excluded s1, s2 and s3 due to their complicated structure (connected with fabella and condyle).

Reconstructed bone images were filtered with an Accurate Gaussian Blur filter (sigma = 0.8) to reduce noise. The real spatial resolution of the image is 2-3 times the pixel size and therefore filtering with a small kernel linear filter does not significantly affect the edge quality. The binarized images were segmented from background using a simple global thresholding method. A fully automated segmentation method was developed for trabecular and cortical shell separation on the basis of previous reports [22-25]. The proposed algorithm was simply based on dilation, connection, erodation, and subtraction, and all procedures were performed using open source software ImgeJ (NIH, http://rsbweb.nih.gov/ij/). The acquired binarized image stack of trabecular bone was analyzed using a CT-Analyser (version 1.6.1). Connectivity density (Conn.D), trabecular bone volume (BV) and surface (BS), bone surface and volume ratio (BS/BV), trabecular number (Tb.N), and trabecular separation (Tb.Sp) were calculated using the Mean Intercept Length (MIL). Trabecular thickness (Tb.Th) was determined using the method of Hildebrand [26]. In addition to the computation of metric parameters, topological parameters were determined so as to describe the 3D nature of the trabecular bone. A trabecular pattern factor (Tb.Pf)), representing the amount of concave (plate-like bone) and convex (rod-like bone) structures was calculated. The higher the Tb.Pf, the more rod-like is the trabecular bone shape. A structure model index (SMI) was measured to determine the prevalence of plate-like or rod-like trabecular structures, where 0 represents "an ideal plate", 3 "a rod or cylinder", and 4 "a sphere". The degree of anisotropy (DA) of a structure, defined as the ratio between the maximal and minimal radii of the MIL ellipsoid, was calculated by superimposing parallel test lines in different directions in the 3D image. DA defines the magnitude of preferred orientation of trabeculae, i.e., the amount of bone that is aligned with the principal axis relative to the other axes. As with pQCT, all microCT measurements and data analyses were performed by the same individual with the CV values being 1.4% for BV, 3.2% for Tb.N, 4.2% for Tb.Th, and 1.2% for Tb.Sp.

### Statistics

Results are expressed as mean (SD). The Shapiro-Wilk test was used to investigate within-group normality for a given parameter of interest. Levene's test was conducted to assess the homogeneity of the variance assumption. The effects of diet (with 2 levels: control and high-fat diet), running (with 2 levels: with and without voluntary wheel running), and their interaction were investigated for each dependent variable using a two-way ANOVA. With the present sample size, the mean statistical power for detecting significant (p < 0.05) diet effect on measured bone traits was 0.88, with four parameters showing power values less than .70 (tBMD = 0.67, CSI = 0.65, Conn.D = 0.60). For significant running effect, the mean power was 0.86, with one parameter showing a value below .70 (tBMD = 0.64). A significant interaction of running by diet was found for CSI with a power of 0.70. The trait means of groups were compared and the significance of differences was determined by post hoc testing using Tukey's HSD. When the normality or equality of variance assumptions was not met, logarithm transformations were conducted. If these parameters still did not meet normality and equality of variance, nonparametric tests were performed and Kruskal Wallis Test was used for multiple comparisons with Chi-Square. The Asymp.Sig level was set at *p *< 0.05. In the Wilcoxon W test for between-group comparisons, the adjusted Asymp.Sig level was set at *p *< 0.008. All the statistical analyses were performed with SPSS 15.0. A *p*-value of < 0.05 was considered significant.

## Results

### Caloric Intake, Body Mass, GTT and ITT

**Figure 2:**
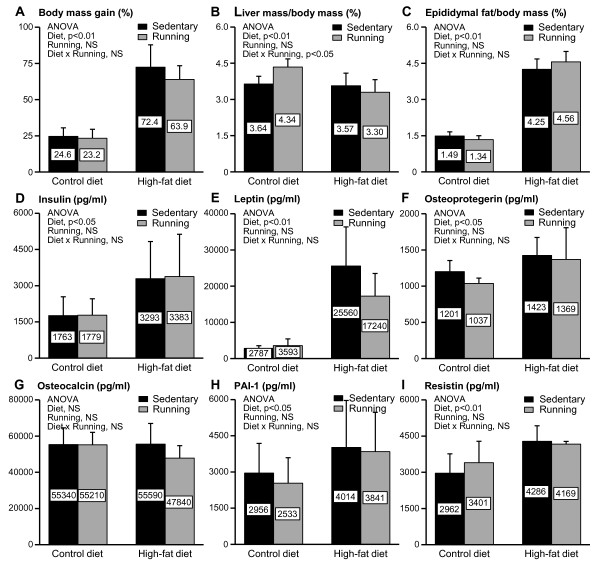
**Body composition and serum biomarkers in mice.** Body mass gain (A), liver mass relative to body mass (B) and epididymal fat pad mass relative to body mass (C) after the 21-week intervention. Insulin (D), leptin (E), osteoprotegerin (F), osteocalcin (G), plasminogen activator inhibitor-1 (PAI-1) (H) and resistin (I) concentrations in the serum samples collected at the end of the 21-week intervention. Means are shown inside the columns. p < 0.05 was regarded as significant (n = 10 mice/group).

Voluntary wheel running increased the total dietary caloric consumption as well as protein and carbohydrate intake. However, the increase was significant only in the mice on the control diet (Table 1). After 4 weeks of intervention, HF and HFR had significantly higher body mass than C and CR. From this time point on, the body mass of mice fed the high-fat diet increased continuously. The body mass of mice fed the control diet reached a peak after 12 weeks of intervention. Finally, the mice in the HF group gained ~72% of body mass and those in C ~ 25% when compared to their initial weight, while the runners gained slightly less body mass, HFR ~64% and CR ~23% (Figure 2A) [21]. Figure 2B shows that the obese mice had significantly lower liver mass relative to body mass than the mice fed with the control diet. Under the control diet, running significantly increased relative liver mass. Consistent with their increased body mass, the obese mice had higher relative epididymal fat pad mass than their normal-weight counterparts (Figure 2C).

**Table 1:** Total energy consumption and the energy derived from fat, protein and carbohydrates during the 21-week intervention, and the results of the glucose and insulin tolerance tests performed at weeks 10 and 18 of the intervention

Basic data	Control diet		High-fat diet		ANOVA (*p *value)		
				
	C (n = 10)	CR (n = 10)	HF (n = 10)	HFR (n = 10)	Diet	Running	Diet*Running
*Energy consumption*							
Total (kcal)	1402 (83)	1513 (76)^a^	1731 (143)^ab^	1771 (132)^ab^	<0.001	<0.05	0.330
Fat (kcal)#	56.1 (3.3)	60.5 (3.1)^a^	1038 (86)^ab^	1063 (79)^ab^			
Protein (kcal)#	259 (15)	280 (14)^a^	346 (29)^ab^	354 (26)^ab^			
Carbohydrate (kcal)#	781 (46)	843 (43)^a^	346 (29)^ab^	354 (26)^ab^			
*GTT and ITT*							
AUC_GTT_10w^†^	1087 (315)	807 (194)^a^	1448 (432)^ab^	1561 (559)^ab^	<0.01	0.510	0.128
AUC_GTT_18w	947 (356)	722 (315)^a^	1527 (216)^ab^	1575 (466)^ab^	<0.01	0.428	0.225
AUC_ITT_10w	778 (105)	727 (155)	980 (159)^ab^	862 (242)^b^	<0.01	0.131	0.540
AUC_ITT_18w	812 (183)	767 (169)	992 (190)^ab^	968 (245)^b^	<0.01	0.586	0.869
Glucose_10w-fasting _(mmol/L)	9.08 (0.68)	8.29 (0.97)^a^	9.74 (0.58)^b^	9.26 (0.92)^b^	<0.01	<0.05	0.547
Glucose_18w-fasting _(mmol/L)	9.26 (1.15)	8.38 (0.92)^a^	10.3 (1.1)^ab^	10.3 (0.9)^ab^	<0.01	0.168	0.197

#Wilcoxon W test for between group comparisons, ^†^Logarithm transformation for ANOVA. ^a^*p *< 0.05 vs C; ^b^*p *< 0.05 vs CR

As expected, the obese mice in HF and HFR had significantly impaired glucose tolerance and insulin sensitivity compared to the mice in C and CR. Voluntary wheel running significantly decreased AUC-GTT in the mice on the control diet, but no significant running-induced changes were found in AUC-ITT. These findings were consistent with fasting plasma glucose levels, which were lower in CR than in C (Table 1).

### Wheel-Running Distance

Both CR and HFR reached their maximum running distance after 4 weeks of intervention, with a gradual decline thereafter. The average daily distances in CR and HFR were 3.48 (1.34) and 3.13 (1.13) km, respectively, with no significant differences between the groups. We have not found any significant differences in the cumulative running distance between CR and HFR, but voluntary exercise training improved the running capacity, especially in the HFR group [21].

### Serum Biomarkers

Diet showed a significant main effect on serum insulin (Figure 2D), leptin (Figure 2E), osteoprotegerin (Figure 2F), PAI-1 (Figure 2H), and resistin (Figure 2I), but not on osteocalcin (Figure 2G). The obese mice had significantly higher levels of these biomarkers compared to the mice fed with the control diet.

### Effects of Diet-Induced Obesity and Voluntary Running on Bone

1) Obese mice had larger and stronger distal metaphysis with more abundant and thicker trabecular bone.

Bone traits measured by pQCT are shown in Table 2. The obese mice had larger bone size (CfB and CSA), higher BMC and higher Imax, Imin, and Ipolar, but lower BMD. Larger traCSA and higher traBMC were also found in the obese mice.

**Table 2:** Cortical and trabecular parameters measured by pQCT from the distal metaphysis of mouse femur dissected after 21-week intervention

pQCT	Control diet		High-fat diet		ANOVA (p value)		
				
	C (n = 10)	CR (n = 10)	HF (n = 9)	HFR (n = 9)	Diet	Running	Diet*Running
CfB (mm)	6.80 (0.15)	6.61 (0.28)	7.19 (0.25)^ab^	7.18 (0.35)^a^	<0.01	0.252	0.291
CSA (mm^2^)	2.85 (0.14)	2.68 (0.18)	3.11 (0.31)^b^	3.16 (0.35)^b^	<0.01	0.491	0.190
BMD (mg/cm^3^)	505 (13)	492 (13)	489 (14)	478 (20)^a^	<0.01	<0.05	0.806
BMC (mg/mm)	1.44 (0.07)	1.32 (0.09)^a^	1.52 (0.11)^b^	1.51 (0.15)^b^	<0.01	0.077	0.114
*Biomechanics*							
Imax (mg·cm)	867 (71)	763 (113)	1025 (124)^ab^	1001 (171)^b^	<0.01	0.129	0.336
Imin (mg·cm)	255 (26)	219 (25)	298 (45)^b^	295 (65)^b^	<0.01	0.179	0.268
Ipolar (mg·cm)	1125 (95)	982 (134)	1323 (164)^ab^	1294 (233)^b^	<0.01	0.120	0.300
CSI × 10^3^(g^2^/cm^4^)	6.77 (0.46)	5.87 (0.55)^a^	6.79 (0.53)	6.81 (0.73)	<0.05	<0.05	<0.05
*Cortex*							
cCSA (mm^2^)	1.23 (0.11)	1.06 (0.09)^a^	1.21 (0.09)^b^	1.17 (0.11)	0.142	<0.01	0.062
cBMD (mg/cm^3^)	779 (11)	757 (23)	770 (19)	752 (31)^a^	0.332	<0.01	0.820
cBMC (mg/mm)	0.95 (0.09)	0.80 (0.08)^a^	0.93 (0.07)^b^	0.88 (0.12)	0.334	<0.01	0.096
cTh (μm)	139 (23)	110 (14)^a^	129 (12)^b^	120 (18)^a^	0.866	<0.01	0.103
*Trabeculae*							
traCSA (mm^2^)	1.63 (0.11)	1.62 (0.12)	1.90 (0.26)^ab^	1.99 (0.32)^ab^	<0.01	0.596	0.522
traBMD (mg/cm^3^)	298 (11)	317 (10)^a^	308 (12)	314 (12)^a^	0.431	<0.01	0.113
traBMC (mg/mm)	0.49 (0.04)	0.51 (0.04)	0.58 (0.08)^a^	0.63 (0.10)^ab^	<0.01	0.121	0.888
mCSA (mm^2^)	0.28 (0.06)	0.29 (0.05)	0.36 (0.10)	0.28 (0.10)	0.261	0.235	0.147

Results are mean (SD) and p < 0.05 was considered as significant. (C = control diet, CR = control diet + voluntary running, HF = 60% fat diet, HFR = 60% fat diet + voluntary running)

^a^*p *< 0.05 vs C; ^b^*p *< 0.05 vs CR

**Figure 3:**
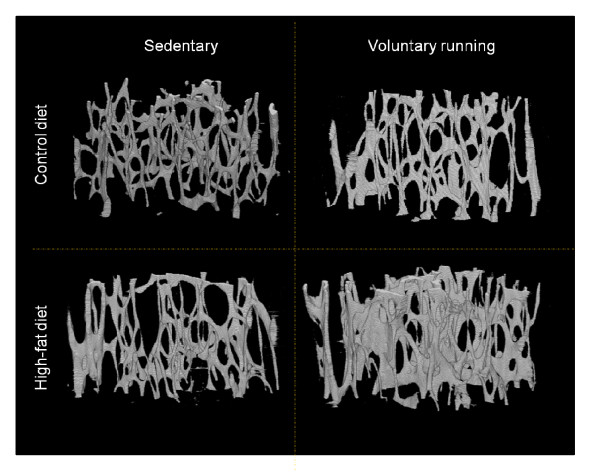
**Representative 3D structure of the distal metaphysis of mouse femur**. A rod-like trabecular structure in animals under control diet with the access to wheel running, but a more plate-like structure in HFR animals was found.

Table 3 shows the 3D trabecular variables measured by μCT at the same site as pQCT. The obese mice had higher BV and BS, a lower BS/BV ratio, higher Tb.Th and Tb.N, smaller Tb.Sp, and higher Conn.D. Consistently with the numerical data, representative 3D images of the μCT scans from the distal metaphysic of HF and HFR animals (Figure 3, lower panel) show that the obese mice have a more abundant, thicker, and well-connected trabecular structure compared to their normal-weight counterparts.

**Table 3:** Trabecular parameters measured by μCT from the distal metaphysis of mouse femur dissected after 21-week intervention.

μCT	Control diet		High-fat diet		ANOVA (*p *value)		
				
	C (n = 8)	CR (n = 9)	HF (n = 7)	HFR (n = 7)	Diet	Running	Diet*Running
Conn.D (mm^-3^)	337 (129)	367 (105)	466 (138)	415 (88)	<0.05	0.810	0.339
BV (10^-1 ^mm^3^)	0.093 (0.015)	0.103 (0.035)	0.112 (0.027)	0.146 (0.034)^ab^	<0.01	0.069	0.333
BS (mm^2^)	1.59 (0.24)	1.70 (0.52)	1.85 (0.39)	2.23 (0.37)^ab^	<0.05	0.106	0.363
BS/BV (mm^-1^)	171 (6)	167 (10)	160 (10)	154 (11)^ab^	<0.05	0.143	0.741
Tb.Pf (mm^-1^)	72.8 (3.4)	69.2 (6.1)	72.1 (7.0)	63.9 (6.2)^ac^	0.165	<0.01	0.260
SMI	2.60 (0.07)	2.54 (0.11)	2.74 (0.12)^b^	2.53 (0.11)^c^	0.089	<0.01	0.061
Tb.Th (μm)	23.7 (1.2)	23.7 (1.2)	26.1 (0.9)^ab^	25.3 (1.7)^ab^	<0.01	0.446	0.388
Tb.N (mm^-1^)	0.288 (0.020)	0.330 (0.112)	0.339 (0.078)	0.429 (0.090)^ab^	0.068	0.158	0.290
Tb.Sp (μm)	429 (4)	428 (9)	418 (10)	414 (13)^ab^	<0.01	0.352	0.627
DA	3.52 (1.49)	3.72 (1.15)	4.08 (1.52)	3.73 (1.36)	0.575	0.883	0.587

Values are mean (SD) and p < 0.05 was considered as significant. (C = control diet, CR = control diet + voluntary running, HF = 60% fat diet, HFR = 60% fat diet + voluntary running)

^a^*p *< 0.05 vs C; ^b^*p *< 0.05 vs CR; ^c^*p *< 0.05 vs HF

2) Voluntary wheel running decreased all the cortical parameters, but increased trabecular mineral density and improved trabecular microstructure.

The runners had smaller cCSA, lower cBMD and cBMC, thinner cTh, but higher traBMD than their counterparts, especially on the control diet (Table 2).

The μCT measurements (Table 3) showed that the runners had smaller Tb.Pf and SMI, indicating a more plate-like structure (Figure 3, right panel). This was more evident in mice fed with the high-fat diet.

A significant interaction between voluntary wheel running and diet on the compressive strength index was found. Under control diet, voluntary wheel running decreased CSI (Table 2).

## Discussion

It is well known that trabecular bone strength is determined not only by the amount of composite material (mineral, protein and water) but also the distribution of these materials (size, area, structural properties). A number of advantages, such as more abundant, thicker, well-connected, and plate-like trabeculae, confer a stronger trabecular bone compartment [27-30]. In the present study we demonstrated that diet-induced obese mice had a larger and stronger femoral metaphysis with more abundant and thicker trabecular bone. Voluntary wheel running decreased all of the measured cortical parameters, but increased trabecular bone mineral density and improved the 3D micro-structure.

Numerous data have shown that obesity is closely associated with dietary fat intake and sedentary life style [31,32]. A close link between body mass and bone mass [17,33] and increased risk for osteoporotic fracture due to low body and thus bone mass [34] has been reported. Our study also found a positive relationship between body mass and bone mass. Increased body mass requires stronger bone; this can be effectively realized through distributing bone mass further from the center of mass rather than dramatically increasing bone density. In our study, although total bone mineral density was decreased to some extent, the enlarged marrow cavity and increased total bone cross-sectional area resulted in a larger and stronger bone as indicated by the increased density-weighted moment of inertia (bending strength). This suggests that endosteal resorption and periosteal formation were enhanced in the obese mice. However, the effects of body mass on the skeleton remain controversial although well documented in obese subjects in previous studies [35,36]. Some studies have shown that obese subjects have weaker bone to bear their over-weight body mass compared to normal counterparts in both humans and animals [37,38]. In our study, after adjusting for body mass (unpublished data), no significant differences in bone traits between the obese and normal weight mice were found. This suggests increased bone strength through enlarged cross-sectional area thus distributing bone mass further from the centre of mass to adapt to the increase in body mass.

In order to further elucidate the effect of obesity and physical activity on bone, we separately estimated the trabecular and cortical bone compartments. It is well known that trabecular bone is the primary target for anabolic or catabolic factors and the most active bone site. The obese mice had larger trabecular area and higher trabecular bone mass than the control mice. In a recent report [39], the authors found increased SMI, decreased Conn.D, and similar Tb.Th in the proximal tibia in diet-induced obese mice. Similarly, we found increased SMI, but increased Conn.D and thicker trabeculae in the distal femur. These discrepancies could be explained by the differences in skeleton sites and our more accurate method of segmentation and higher resolution (2.8 μm), which could preserve natural structure and detect even tiny connections. The cortical parameters studied (cCSA, cBMD, cBMC, and cTh,) were not significantly influenced by diet-induced obesity, as also found in a previous study [39]. However, some studies have shown positive effects of diet-induced obesity on cortical bone size [40], while negative effects on cortical bone mass [41] and size-independent mechanical properties [40] have also been suggested. These controversial results may be due to differences in animal age and the skeleton site measured as well as different measuring techniques.

Bone is a dynamic structure monitored by both intrinsic (body mass, hormone, cytokine, and other intrinsic factors) and extrinsic factors (environmental factors including physical activity, life style, etc.). On the one hand, the effect of diet-induced obesity on bone could be explained by alteration in the intrinsic mechanical loading environment caused by the increase in body mass. On the other hand, adipose tissue is regarded not just as a passive tissue for the storage of excess energy in the form of triglycerides, but also as an active endocrine organ secreting a variety of biologically active molecules, for example, leptin [42], resistin [43] and adiponectin [44]. A cascade of events such as intense conversion of androgens into estrogens occurring in adipose tissue, alterations in other hormones or cytokines, and hyperinsulinemia may influence the bone microenvironment and increase bone mass [17]. The elevated plasma leptin level in diet-induced obesity is a predictor of body mass accrual in different species [12,45,46]. The serum leptin level also regulates bone mass [47]. However, the results of published studies on the effects of leptin level on bone are complex and controversial [42]. Resistin is a controversial inflammatory-related factor [48], which also influences both osteoclast and osteoblast activity, resulting in increased bone remodelling [43]. We found higher plasma leptin and resistin levels in obese mice, suggesting that plasma leptin and resistin levels have a positive effect on bone. However, their effects on bone mass and strength were the opposite, bone mass and strength showing a positive association with plasma leptin level and a negative association with resistin level (unpublished data). However, increased bone mass with increased body mass independent of leptin was also reported in a recent study [33]. So far, the mechanism of interaction between bone metabolism and resistin remains unclear.

The development of obesity is associated with chronic inflammatory status, coinciding with significantly increased macrophage infiltration in adipose tissue and the expression of inflammatory cytokines, such as TNF-α, IL-6, monocyte chemotactic protein-1, and plasminogen activator inhibitor type-1 (PAI-1) [6]. All of these inflammatory reactions are considered to be responsible for the majority of the obesity-related syndromes. Not surprisingly, this inflammatory status also influences bone metabolism through altering the micro-environment surrounding the bone cells. Over expression of PAI-1 increased bone strength and mineralization in an age- and gender-specific manner [49]. Here, we found that a higher level of PAI-1 in obese mice correlated significantly with trabecular thickness, suggesting that PAI-1 had a positive effect on bone. In addition to these altered adipokins and inflammatory factors, we also detected higher levels of osteoprotegerin that is a bone resorption inhibitor [50]. Together with the aforementioned factors, higher body weight may increase bone strength by shifting bone remodelling towards more active bone formation.

In order to investigate possible intervention methods, we also examined the effects of voluntary exercise. Here we found that voluntary wheel running was associated with a non-significant reduction in body mass with a concomitant improvement in glucose tolerance and insulin sensitivity and an increase in relative liver mass. Further studies are needed to find out whether the relative increase in liver mass in runners is associated with factors such as increased protein synthesis or glycogen storage. The minor reduction in body mass was not due to reduction in dietary intake. In fact, the runners consumed slightly more energy than their sedentary counterparts. Thus, the reduction in body mass was secondary to the increase in exercise-associated energy expenditure.

Physical activity has been shown to associate with bone mass and strength and to have positive effects on bone properties [51,52]. In obese subjects, physical activity increased total, hip, and lumbar bone mineral content [53] and decreased plasma leptin level [54,55]. More excitingly, long-term leisure time physical activity also showed positive effects on both cortical thickness and trabecular bone after controlling for the subjects' genetic background [20]. The present study showed that voluntary exercise increased trabecular bone mineral density and improved the bone geometrical structure but led to a decrease in all of the cortical parameters. Previous animal studies have also shown positive effects of exercise on bone in different species at different ages with different types of exercises [56-59]. Most of these studies have focused on either cortical or trabecular bone mass or strength, with very few studies reporting micro-structural alteration induced by exercise. In C57BL/6J mice, from the age of six weeks onwards, trabecular volume and trabecular number are generally decreased [60] while trabecular thickness and trabecular space are increased up to an age of 24 weeks [61]. Mori et al. [62] showed that intermittent voluntary climbing in eight-week-old C57BL/6J mice increased trabecular bone volume and reduced bone resorption, partially due to initial down-regulation of marrow osteoclastogenic cells and up-regulation of osteogenic cells, while further exercise desensitized them. In our study, the voluntary running lasted for 21 weeks, thus covering the entire growth period. We found that voluntary exercise tended to increase trabecular bone volume and decreased trabecular pattern factor and structure model index, shifting trabecular bone towards a stronger, more plate-like structure. However, in agreement with our previous report [21], we found that voluntary exercised animals under control diet had lower total BMC and cortical parameters. Similar findings were also reported in the rat tibia after intensive treadmill running [63], in the mice tibia after weight-bearing running during growth [64], and in 23-week-old female C57BL/6J mice after one month of voluntary wheel running [65]. These effects might be explained by overactive modeling or remodeling of bone under continuous mechanical stimulation during growth [66-68]. However, histo-morphometric analyses [63] have suggested that decreased osteoblastic activity rather than a global adaptation of bone remodeling resulted in reduced longitudinal bone growth and bone loss in young rats under strenuous training. Another possible reason may be exercise-induced weight loss, which is accompanied by a reduced mechanical strain on the skeleton and decreased need of strong bones. Consequently, although numerous data indicate that, during growth, physical activity imposes its effect on bone more efficiently, the exercise programs or activities that will optimize bone structure and strength still remain unclear [18].

## Conclusions

Diet-induced obesity had positive effects on total bone mass and strength rather than on total BMD, and also positive effects on trabecular structure, whereas no effects on cortical parameters were noted. Voluntary training had protective effects on trabecular bone mineral density and 3D microstructure while limiting the overall growth of cortical bone. Voluntary training combined with dietary intervention showed more apparent effects on trabecular microstructure. These results suggest that both diet and voluntary exercise affect bone properties in a site-specific manner and that the interaction between physical activity and diet is highly complicated.

## Competing interests

The authors declare that they have no competing interests.

## Authors' contributions

HM, MS, HK, PR, and HS contributed to the experiment design. HM, MS, ST, RT, HK, and PR participated in data collection. HM, TT, and JT contributed to the microCT measurement and analysis. HM and PR performed statistical analyses, interpreted the data, and drafted the manuscript. HS critically reviewed the paper. All authors read and approved the final manuscript.
